# Assessing the difficulty of annotating medical data in crowdworking with help of experiments

**DOI:** 10.1371/journal.pone.0254764

**Published:** 2021-07-29

**Authors:** Anne Rother, Uli Niemann, Tommy Hielscher, Henry Völzke, Till Ittermann, Myra Spiliopoulou

**Affiliations:** 1 Faculty of Computer Science, Otto von Guericke University Magdeburg, Magdeburg, Germany; 2 Institute for Community Medicine, University Medicine Greifswald, Greifswald, Germany; Universidad de Granada, SPAIN

## Abstract

**Background:**

As healthcare-related data proliferate, there is need to annotate them expertly for the purposes of personalized medicine. Crowdworking is an alternative to expensive expert labour. Annotation corresponds to diagnosis, so comparing unlabeled records to labeled ones seems more appropriate for crowdworkers without medical expertise. We modeled the comparison of a record to two other records as a triplet annotation task, and we conducted an experiment to investigate to what extend sensor-measured stress, task duration, uncertainty of the annotators and agreement among the annotators could predict annotation correctness.

**Materials and methods:**

We conducted an annotation experiment on health data from a population-based study. The triplet annotation task was to decide whether an individual was more similar to a healthy one or to one with a given disorder. We used *hepatic steatosis* as example disorder, and described the individuals with 10 pre-selected characteristics related to this disorder. We recorded task duration, electro-dermal activity as stress indicator, and uncertainty as stated by the experiment participants (*n* = 29 non-experts and three experts) for 30 triplets. We built an Artificial Similarity-Based Annotator (ASBA) and compared its correctness and uncertainty to that of the experiment participants.

**Results:**

We found no correlation between correctness and either of stated uncertainty, stress and task duration. Annotator agreement has not been predictive either. Notably, for some tasks, annotators agreed unanimously on an incorrect annotation. When controlling for Triplet ID, we identified significant correlations, indicating that correctness, stress levels and annotation duration depend on the task itself. Average correctness among the experiment participants was slightly lower than achieved by ASBA. Triplet annotation turned to be similarly difficult for experts as for non-experts.

**Conclusion:**

Our lab experiment indicates that the task of triplet annotation must be prepared cautiously if delegated to crowdworkers. Neither certainty nor agreement among annotators should be assumed to imply correct annotation, because annotators may misjudge difficult tasks as easy and agree on incorrect annotations. Further research is needed to improve visualizations for complex tasks, to judiciously decide how much information to provide, Out-of-the-lab experiments in crowdworker setting are needed to identify appropriate designs of a human-annotation task, and to assess under what circumstances non-human annotation should be preferred.

## Introduction

Crowdsourcing is an approach where the wisdom of the crowd is used to solve a specific problem [1]. Crowdsourcing tasks and their annotation are becoming popular in health and medical research [2, 3]. Some approaches focus solely on expert knowledge [3–5], while others involve the general population [2, 6–8]. Application fields for crowdsourcing in medical research include the tracking and tracing of outbreaks [6, 7], evaluation of radiological images [9, 10] or structural prediction in molecular biology [11, 12].

In this study, we investigate the potential of crowdsourcing to assess whether an individual is more similar to a healthy or a diseased individual using hepatic steatosis as outcome. The assignment of a label directly to the individual would correspond to a medical diagnosis, i.e. a task that demands medical expertise. We therefore resort to a different paradigm for the annotation, namely similarity-based annotation using *triplets*. The annotation task is that of showing three objects (i.e. one ‘triplet’), two of known and opposite labels and one of unknown label, and asking each annotator to assign this third object to the most similar of the two labeled ones. Showing triplets emanates from the insight that “For humans, it is usually easier to make statements about the similarity of objects in relative, rather than absolute terms” [13]. Although there are experimental investigations on triplet-based annotation for images, see e.g. [14], there are (to the best of our knowledge) no investigations on how crowdworkers would perform in the triplet-based annotation task in the medical context. This implies that models of crowdworkers, as proposed e.g. in [15], cannot be used, since there is no a priori knowledge on the task complexity ‘from a purely objective standpoint’, i.e. from ‘the characteristics of the task alone’ (quoting from [15], preample of section 3.1). Our experimental study deals with this issue by investigating the interplay between performance and uncertainty for a triplet-based annotation task, when the objective task difficulty cannot be known in advance.

Hepatic steatosis is defined as a fat deposit >5% in the liver [16] and develops from increased hepatic lipid storage [17]. The prevalence of hepatic steatosis was reported to be about 25% worldwide [18]. The aim of our study was to evaluate whether students of computer science were able to identify individuals with and without hepatic steatosis based on a visualization of the risk factors age, sex, alanine-aminotransferase (ALAT), low-density lipoproteine (LDL) cholesterol, alcohol consumption, hypertension, beta-blocker intake, type 2 diabetes mellitus, smoking status, and c-reactive protein (CRP). The data was extracted from the population-based “Study of Health in Pomerania” [19]. The results of the students were compared to those of three experts from the fields of medicine, epidemiology and data visualization.

Based on that data, we investigated the relationship between annotator uncertainty and performance for triplet annotation tasks. More precisely, we investigated the following questions:

Q1 How to assess whether annotators feel uncertain about their decisions?
Q2 How does uncertainty associate with performance?
Q3 How does agreement associate with performance?
Q4 How to explain the relationship between uncertainty and performance?

The uncertainty of an annotator may be high because of the annotator’s expertise [20] or because of the inherent difficulty of the task to be performed [21]. The self-estimation of this uncertainty may be affected by the choice of representation, e.g. by the number of offered categories. Hence, next to stated uncertainty, we investigate stress levels, as recorded through electrodermal activity, as well as task duration as three means of expressing uncertainty among the annotators (Q1).

We further defined quantifiable measures for *performance* and *agreement* among annotators, and used them for Q2 and Q3. To explain the relationship between uncertainty and performance, we designed a machine learning utility that receives the same inputs as human annotators and then annotates the data on the basis of a mechanistic and thus objective, quantifiable measure of similarity. This “Artificial Similarity-Based Annotator” (ASBA) serves as basis for Q4, thereby establishing a link between the (observable) performance of the annotators and the (unobservable and unknown) inherent difficulty of the annotation task.

Since the experiment participants were unfamiliar with the annotation task, we anticipated the likelihood of different uncertainty and agreement at the beginning and at later stages of the experiment. Therefore, we also investigated the following question:

Q0 To what extent do annotators undergo an *acclimatization phase* characterised by differences in individual performance and uncertainty and in differences in the likelihood of disagreement?

## Materials

We designed an annotation experiment for the annotation task of *deciding the class of an medical record on the basis of its similarity to two medical records of known class membership*. The experiment was approved by the “German Association for Experimental Economic Research e.V.” with Institutional Review Board Certificate No. NI4HLBHn. The data of the experiment participants were analysed anonymously. All experiment participants gave informed written consent.

The data to be annotated came from the population-based study SHIP. All SHIP-study participants gave informed written consent. The SHIP study followed the recommendations of the Declaration of Helsinki. Approval for SHIP was given by the local Ethics Committee of the University of Greifswald (No. BB 39/08). In the following, we use the term ‘annotator’ to refer to the 29 non-expert experiment participants and not to the participants of the SHIP-study, whose assessments we used in the annotation tasks. When we refer to the three experts involved in the experiment, we use the term ‘expert annotator’ or ‘expert’ for short. We use the terms ‘experiment participant’ and ‘annotator’ (expert, non-expert, as appropriate) as synonyms.

### Experiment design

**Fig 1:**
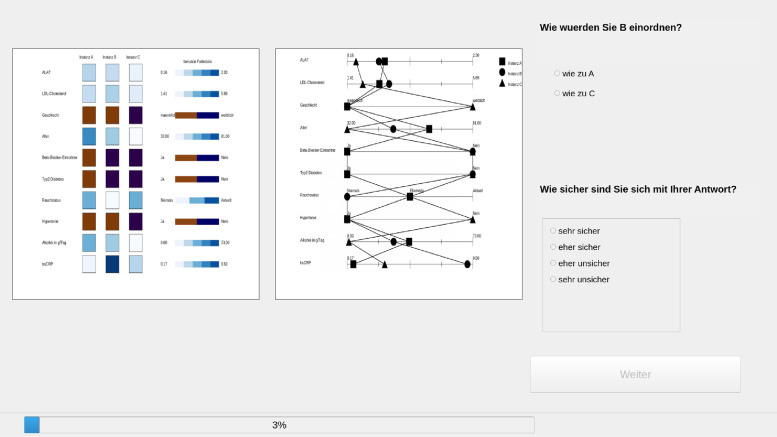
Interface of the annotation experiment. Leftmost white box: tile-based configuration; white box in the middle: line-based configuration. Furthermore, questions are asked about the similarities of records A and C in comparison to B and the uncertainty of the answer.

Each experiment participant of our experiment was asked to annotate 30 records incorporated in triplets, i.e. perform 30 annotation tasks and each record was described by a fixed set of 10 variables (Table 1). These 10 variables comprise well-known predictors for hepatic steatosis including age, male sex, metabolic markers, inflammatory markers as well as smoking, alcohol consumption and medication intake [22]. Fig 1 shows a screenshot for one of the triplets as it was presented to the experiment participant: the record to be assigned to a class is the middle one, marked as column “B”, while the records of known class membership are marked as columns “A” and “C”. The experiment participant was shown two representations, a tile-based one (leftmost box) with the numerical values marked as shades, and a lines-based one (box in the middle), where the position of the middle record’s value for some variable indicates its distance from the values of the variable for the other two records. The names of the variables refer to properties of the records, and are listed in Table 1.

**Table 1:** Overview of used variables in the annotation experiment.

variable	description	values margin
age	age at examination	years
alat_s	alanin-aminotransferase	μkatal/l
alcohol in g/day	alcohol in g/day	g/day
beta blocker	beta blocker intake	yes or no
crp_hs	high-sensitive CRP	mg/l
diabetes	type 2 diabetes mellitus	yes or no
hypertonia	hypertension	yes or no
ldlch	LDL-cholesterol	mmol/l
sex	sex	male or female
smoke_status	smoking status	former, never or current
livfat_per	liver fat concentration	%
stea	hepatis steatosis	0 or 1

For each triplet experiment participants were asked two questions. One concerned the class membership itself and was formulated as (cf. Fig 1, right uppermost part): “How would you classify B?”. The second question concerns the certainty of the experiment participant in annotating the triplet (cf. Fig 1, right lowermost part) as one of the four discrete options “very certain”, “rather certain”, “rather uncertain”, and “very uncertain”.

The triplets of the data set used for the annotation experiment were selected from 852 individuals of the “Study of Health in Pomerania” (SHIP-2), the second follow-up of a population-based study in Northeast Germany. Hepatic steatosis was used as classification outcome [19]. Hepatic steatosis was defined according to liver fat measurements based on magnetic resonance imaging (MRI) [23]. Ninety records were selected based on the MRI-assessed fat fraction in the liver.

Selection of the 90 records in the 30 triplets was done as follows. The 852 individuals of SHIP-2 were categorized into three classes: “no hepatic steatosis” (liver fat fraction ≤ 5.0%, n = 501), “mild hepatic steatosis” (5.0% ≤ liver fat fraction <14%, n = 238), and “moderate to severe hepatic steatosis” (liver fat fraction ≥ 14%, n = 113) [24]. We randomly selected 45 individuals from the class “no hepatic steatosis” and 45 from the class “moderate to severe hepatic steatosis”. Each of these two subsamples was further split into three groups of 15 individuals: groups 1, 2 and 3 consisted of individuals without hepatic steatosis, while groups 4, 5 and 6 consisted of individuals with hepatic steatosis. The individuals inside each group were numbered from 1 to 15. Then, 15 triplets were built by selecting individuals with the same number (which served as identifier) from groups 1, 2 and 6, and further 15 triplets were built similarly from the groups 3, 4 and 5. This means that the triplets from groups 1, 2 and 6 contained two individuals without hepatic steatosis and one with, while the triplets from groups 3, 4 and 5 contained two individuals with hepatic steatosis and one without.

The true class membership of records inside a triplet was not shown to the experiment participants. Rather, each triplet appeared on the annotation screen to be processed. Record A (leftmost one) was always an individual with hepatic steatosis, record C was always an individual without hepatic steatosis, and record B had or had not the disorder depending on whether the triplet came from groups 1, 2 and 6 or rather 3, 4 and 5. The first 10 variables listed in Table 1 were shown to the experiment participants for each entry. The last two variables, below the horizontal line, were not shown to the experiment participants. The variable livfat_per depicts the fraction of fat in the liver and was used for the class separation. The last row determines the class, i.e. “moderate to severe hepatic steatosis” ≡ 1 and “no hepatic steatosis” ≡ 0.

For a given triplet, class membership may thus depend on (dis)similarity between record B and the records B and C with respect to one, two or all of the variables. Since this is not known in advance, there is no ground truth on the *objective difficulty* of annotating the triplets. To tackle this challenge in the context of Q4, we introduce a mechanistic annotator, as explained under ‘Methods’ for Q4.

### Experiment participants and data recorded for them

The experiment was performed by n = 29 non-expert annotators (10 female, 19 male; average age: 24 years) and by three expert annotators.

The non-expert annotators were recruited among university students, mostly from the faculty of computer science (25 out of 29 experiment participants). Exclusion criteria were medical expertise on the disease presented as part of the annotation task (hepatic steatosis) and colour blindness. With the first exclusion criterion we excluded volunteers who would have been able to perform a medical diagnosis on each of the records A, B and C separately and thus annotate record B without considering similarity. With the second exclusion criterion we excluded volunteers who might have been unable to discern the color shades for some of the variables and thus fail to identify similarities among values.

Among the three expert annotators, Expert 1 (author TI) was very familiar with the dataset, and has performed the selection of characteristics and the sampling from SHIP-2. Expert 2 (author UN) was very familiar with the dataset. Expert 3 was a physician, who was not familiar with the dataset and was not aware that the experiment was about hepatic steatosis. So, all of them were able to identify variables that were more important for the annotation than others. However, none of them was skilled in doing a medical diagnosis for each of the participants separately, not even Expert 3 since he did not know what disorder was considered. Thus, the first exclusion criterion used for the non-experts was also satisfied by the expert annotators. None of the three experts was color-blind, so the second exclusion criterion was also satisfied. The annotations of the experts were used as reference with respect to uncertainty and correctness in the assignment of records to a class.

Next to the “Collection of Experiment Data” described below, the experiment participants filled an end-of-study questionnaire with items on sociodemographics, level of IT expertise and background knowledge, as well as feedback on the experiment. Data on country of origin and mother tongue were collected to assess whether an experiment participant was more likely to read from the right to the left or vice versa, and whether an experiment participant might have difficulties in understanding the semantics of the variables. Twenty-three of 29 experiment participants had German as mother tongue. Out of 29 experiment participants, 25 were right-handed, the remaining 4 were left-handed. Data on handedness and mother tongue were not further considered in the present investigation.

According to the end-of-study questionnaire, the expertise of the non-expert annotators in medicine, data mining and image processing were as shown on Table 2.

**Table 2:** Expertise of the 29 experiment participants in medicine, data mining, image processing.

Background of non-experts	Medicine	Data Mining	Image processing
none	17 (58.62%)	11 (37.93%)	11 (37.93%)
little	10 (34.48%)	15 (51.72%)	13 (44.83%)
much/very much	2 (6.90%)	3 (10.34%)	5 (17.24%)

### Collection of experiment data

During the experiment, each experiment participant had to annotate the record B for a total of 30 triplets, and also report on his/her perceived uncertainty concerning the annotation of the record. Furthermore, the duration of processing each triplet was recorded, as well as the electrodermal activity during the experiment.

For recording of the electrodermal activity, EDA (unit *μ*S), we used the EDA and Activity Sensor “EdaMove 4” from movisens GmbH. According to the information given by the experiment participants on handedness (right hand or left hand), the sensor was fixed on the other hand.

The data of the “EdaMove 4” sensors were collected with the “DataAnalyzer” (movisens GmbH). Then, we marked the beginning and the end of the experiment manually, and used the processing duration per triplet (which was recorded) to segment the whole time series of each experiment participant into one segment per experiment participant and triplet. This segment contained the electrodermal activity of the experiment participant during the annotation of the triplet. We refer to these segments as *time series* thereafter.

The experiment was performed in the Experiments Lab of the faculty of computer science of the Otto-von-Guericke University of Magdeburg, Germany. During the experiment, there was always an experiment supervisor in the room. The laboratory room was cooled to 23 degrees Celsius.

## Methods

To address the research questions, we modelled *correctness* and *uncertainty*. We designed a machine learning method that annotates the data on similarity, and we compared its correctness and uncertainty to that of the experiment participants. We used heatmaps to visualize the interplay of uncertainty indicators to correctness and we applied mixed models to assess statistical significance. We describe these methods hereafter.

### Quantifying performance as correctness

We defined “correctness” as the number of correct answers returned by the experiment participants (the volunteers playing the role of “crowdworkers”, denoted as “annotators” hereafter), i.e. the number of triplets where the middle record was correctly classified. We distinguished between:

- “annotator correctness” *A_correctness*(*x*) counted for an annotator *x* over all triplets
- “triplet correctness” *T_correctness*(*t*) counted for a triplet *t* over all annotators, and intended to identify triplets that are potentially more difficult to annotate

We derived *A_CorrectnessRatio*(*x*) and *T_CorrectnessRatio*(*t*) by dividing *A_correctness*(*x*) by the number of triplets, i.e. 30, and accordingly dividing the *T_correctness*(*t*) by the number of annotators (29 non-experts, resp. 3 experts).

### Modelling uncertainty (Q1)

We considered three indicators of uncertainty, namely “stated uncertainty”, as perceived by an annotator for a triplet, “duration” on the basis of time needed for the annotation of each triplet and stress levels on the basis of electrodermal activity (“eda” or “EDA”). To make clear that we do not refer to stress as a medical condition but rather as an observable quantity, we prefer the term “eda” to the term “stress level”, and use the latter only when the context is clear.

#### Uncertainty as stated

We quantified the “stated uncertainty” *Stated_U*(*x*, *t*) of an annotator *x* for triplet *t* on the basis of the answer of *x* to the “How certain” question for *t*. We encoded the four possible answers into following values: 0 (very certain), 1 (rather certain), 2 (rather uncertain) and 3 (very uncertain), i.e. higher values for higher uncertainty. Then, similarly to correctness, we defined:

- “Annotator Stated_U” *A_Stated_U*(*x*) = ∑_*t*_ *Stated_U*(*x*, *t*), i.e. the sum of Stated_U values marked by annotator *x* over all triplets
- “Stated_U towards triplet” *T_Stated_U*(*t*) = ∑_*x*_ *Stated_U*(*x*, *t*), i.e. the sum of Stated_U values marked by the annotator for triplet *t*

We derived *A_Stated_U_Ratio*(*x*) by dividing *A_Stated_U*(*x*) by the number of triplets, and *T_Uratio*(*t*) by dividing *T_Stated_U*(*t*) by the number of annotators.

Finally, we derived *binary Stated_U* scores and ratios by aggregating the two original Stated_U values 0 and 1 into 0 for “low Stated_U” and the two original Stated_U values 2 and 3 into 1 for “high Stated_U”. From these, we derived the binary counterparts of *A_Stated_U* and *T_Stated_U* and the corresponding ratios.

#### Uncertainty indicators: Task duration and EDA

We investigated ways of assessing uncertainty and designed two indicators of uncertainty. For an annotator *x* with respect to triplet *t* we captured following indicators:

- *duration*(*x*, *t*): elapsed time needed by *x* to annotate triplet *t*
- *eda*(*x*, *t*): mean of the electrodermal activity values recorded by the DataAnalyzer [25] for *x*, when sampled at 10-second intervals during *duration*(*x*, *t*)

Both indicators are affected by personal traits of the annotators. Some annotators may be slower in their annotations than others. Electrodermal activity depends on physiology, hence some annotators may exhibit higher activity recordings than others. Moreover, the number of 10-second samples for an annotator *x* varies with the triplet *t*, so that some mean values of *eda*(*x*, ⋅) are computed over many samples and others over few samples or one sample only.

### Associating correctness with uncertainty (Q2)

To highlight the relationship between correctness and each of the three indicators of uncertainty, namely Stated_U, eda and duration, we used mixed models [26, 27] with random intercept and for statistical significance, we set *p* < 0.05.

Since Stated_U is widely used in crowdsourcing as indicator of true uncertainty, we also used visualizations to show this relationship. We built a two-coloured heatmap of annotators (rows) and triplets (columns): intense colours indicate low Stated_U, faint colours indicate high Stated_U; a blue colour (intense or faint) indicates incorrect annotation, green colour (intense or faint) indicates correct annotation. We expected that triplets/columns annotated with high Stated_U would have a mix of blue and green cells, since annotators that felt uncertain of their answer would choose the correct and the incorrect answer with equal probability. Similarly, we expected that triplets/columns annotated with low Stated_U would consist mostly of green cells, i.e. that annotators would converge to the correct answer. Hence, we expected that faintly coloured columns would not have a dominant colour and that intensely coloured columns would have green as the dominant colour.

Next to visualizations for the inspection of the uncertainty/correctness relationship, we investigated the statistical significance of the relationship. We applied mixed models with random intercepts to identify associations between correctness and the three uncertainty quantifications. We skipped the first three triplets because of the effects of the acclimatization phase on duration, one of the three indicators.

### Measuring agreement (Q3)

We computed the *agreement* among annotators with respect to a triplet *t* by first counting the annotators who considered the middle instance of *t* as similar to the left instance A, *voteFor*(*t*, *A*), and the annotators who opted for the right instance C instead, *voteFor*(*t*, *C*).

In crowdsourcing, it is expected that the likelihood of correct annotation increases with the agreement quantity [20]. We investigated this postulation under Q3. To this purpose, we first computed Krippendorff’s *α* [28] as an overall indicator of agreement among the annotators for the triplet annotation task as a whole, and considered the guideline of [29] about the thresholds for *α*, namely that it should be above 0.800 for reliable results though values as low as 0.667 might also be tolerated (page 429 of [29], third item of recommendations).

We then quantified the amount of agreement *agrm*(*t*) for each triplet separately as normalized majority:

$$\begin{array}{c} \text{agrm}\left(t\right)=\frac{1}{n}\max\left\{\text{voteFor}\left(t,A\right),\text{voteFor}\left(t,C\right)\right\} \end{array}$$ (1)

The valuerange of this quantity is [0.5, 1], with lower values indicating less agreement. We mapped this quantity into a binary indicator of agreement subject to a threshold *τ* ∈ (0.5, 1] as:

$$\begin{array}{c} {\text{agreement}}_{\tau }\left(t\right)=\{\begin{array}{cc} 1 & \text{iff}\text{agrm}(t)\geq \tau \\ 0 & \text{otherwise} \end{array} \end{array}$$ (2)

We started with a conservative discretization, by using a threshold $\tau =\frac{2}{3}$. Then, we used a more restrictive threshold $\tau =\frac{3}{4}$. We used these thresholds to also capture the overall attitude of the *n* annotators towards a triplet. In particular, for each triplet *t*, we mapped the correctness towards triplet *T_correctness*(*t*) and the stated uncertainty towards triplet into binary values, subject to *τ* as follows:

$$\begin{array}{c} T\_\text{correctness}\_{\text{Binary}}_{\tau }\left(t\right)=\{\begin{array}{cc} 1 & T\_\text{correctness}(t)\geq \lfloor n\times \tau \rfloor \\ 0 & \text{otherwise} \end{array} \end{array}$$ (3)

and

$$\begin{array}{c} T\_\text{Stated}\_U\_{\text{Binary}}_{\tau }\left(t\right)=\{\begin{array}{cc} 1 & T\_\text{Stated}\_U(t)\geq \lfloor n\times \tau \rfloor \\ 0 & \text{otherwise} \end{array} \end{array}$$ (4)

### Linking uncertainty to correctness with help of the Artificial Similarity-Based Annotator—ASBA (Q4)

To increase the understanding of the relationship between correctness and uncertainty, we would ideally consider the *objective difficulty* in annotating each triplet as a confounder. Since annotation of triplets is a new task, there is no ground truth on how difficult each triplet is. Therefore, we compared annotator correctness to that of a deterministic software—the Artificial Similarity-based Annotator (denoted as ASBA hereafter). We designed ASBA to take each triplet as input and to return the nearest neighbour of record B. Being a piece of software, ASBA can solve numerical computations of similarity much better than the human eye. This allowed us to use ASBA as gold-standard for the role of Triplet ID on correctness and uncertainty, in the sense that if the records were very similar in *all* variables and if all variables taken together were inadequate for a decision, then ASBA would fail. If less variables were necessary, then the human experts could be better than ASBA.

For the nearest neighbour (1NN) computation in ASBA, we used the Heterogeneous Euclidean Overlap Metric (HEOM), cf. [30] as distance measure between two records, which aggregates distances between continuous variables and between nominal variables. We applied HEOM on the ten variables listed in Table 1, which were shown to the annotators.

To each continuous variable *Y* with range [min_*Y*_, max_*Y*_] we applied the Euclidean distance after normalizing each value *y* into $\frac{y-\min_{Y}}{\max_{Y}-\min_{Y}}$ using the 90 records of the 30 triplets.

To simulate uncertainty, we defined ASBA_U for a triplet *t* as:

$$\begin{array}{c} ASBA\_U\left(t\right)=\frac{\text{smallest}\_\text{distance}(t)}{\text{largest}\_\text{distance}(t)} \end{array}$$ (5)

where *smallest_distance*(*t*) = min{*HEOM*(*t*.*A*, *t*.*B*), *HEOM*(*t*.*C*, *t*.*B*)} and *largest_distance*(*t*) = max{*HEOM*(*t*.*A*, *t*.*B*), *HEOM*(*t*.*C*, *t*.*B*)}, and *t*.*A*, *t*.*B*, *t*.*C* denote the records A, B and C for triplet *t*.

We then discretized ASBA_U into the same four values 0 (very certain), 1 (rather certain), 2 (rather uncertain) and 3 (very uncertain) by rounding:

$$\begin{array}{c} \text{ASBA}\_U\_\text{Aggregated}\left(t\right)=\text{round}(\frac{ASBA\_U(t)}{3}) \end{array}$$ (6)

Finally, we discretized ASBA_U subject to a boundary *τ*_*ASBA*_ as:

$$\begin{array}{c} ASBA\_U\_{\text{Binary}}_{\tau_{ASBA}}\left(t\right)=\{\begin{array}{cc} 0 & ASBA\_U\left(t\right)<\tau_{ASBA}\\ 1 & \text{otherwise} \end{array} \end{array}$$ (7)

indicating that when $ASBA\_U\_{\text{Binary}}_{\tau_{ASBA}}\left(t\right)=0$ ASBA is very certain, while $ASBA\_U\_{\text{Binary}}_{\tau_{ASBA}}\left(t\right)=1$ means that ASBA is very uncertain. It should be stressed that ASBA is a deterministic mechanism and so is its uncertainty.

### Highlighting the role of Triplet ID on the interplay between uncertainty and correctness (Q4)

The Triplet ID has two aspects: it captures the order in which the triplets were shown to the annotators, and it expresses the unique content of the triplet. By its design, ASBA fully concentrates on the contents of the triplets and is insensitive of the ordering of the triplets. The annotators may be affected by the ordering of the triplets, though. Thus, we placed the statistical analysis of the dual role of Triplet ID on annotator correctness and uncertainty after the juxtaposition of annotators and ASBA, and used this juxtaposition to interpret the results of the statistical analysis.

## Results

To address the research questions, we studied the behaviour of the annotators across the dimensions of correctness, Stated_U, EDA and duration of annotation.

### Q0: The acclimatization phase only affects the duration of the annotation tasks

**Fig 2:**
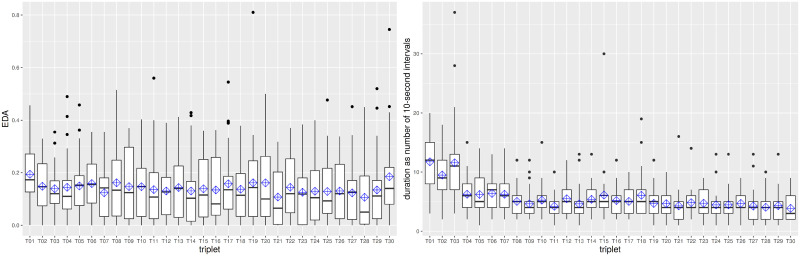
Boxplots of EDA (left subfigure) and of duration (right subfigure) with one box per triplet. The order of the triplets did not have any evident effect on EDA but had an effect on duration.

We defined the *acclimatization phase* as the number of annotations at the beginning of the experiment, during which the *observed* behaviour of the annotators was different than later on. The two dimensions of *observed behaviour* are shown in Fig 2, where we depict one boxplot per triplet containing the EDA (left subfigure) and the duration (right subfigure) for each annotator as a dot inside each box. There is no substantial difference in the EDA of the annotators (left subfigure), but there is substantial difference in the duration (right subfigure). Hence, *there is* an acclimatization phase and it encompasses *3 triplets*.

**Fig 3:**
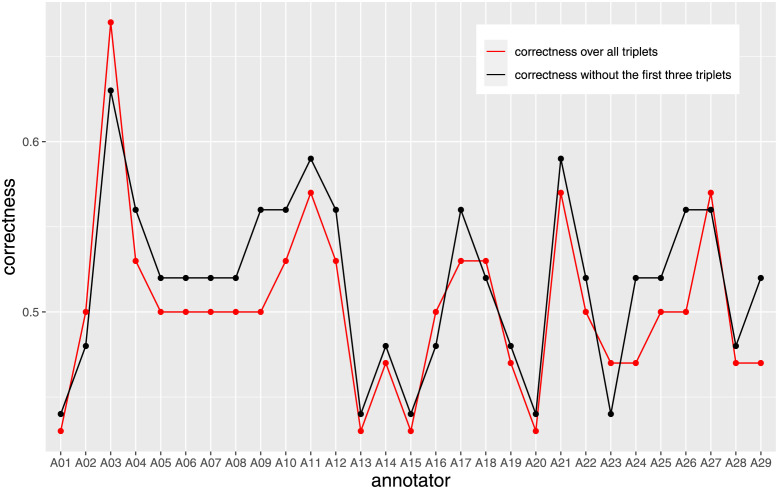
*A_CorrectnessRatio* for the 29 non-expert annotators, when all triplets are considered (red line) and when the first three are removed (black line). Although a slight improvement can be perceived for most of the annotators, the difference is very small, implying that the acclimatization phase has almost no effect on correctness.

For each annotator we captured the correctness achieved over all triplets (red line) vs all but the first three triplets (black line): the correctness for 23 of 29 annotators was slightly better when the first three triplets were excluded, but overall the values were very low (between 0.4 and 0.6) and the individual differences mostly very small between the two scenarios (Fig 3).

Hence, the effect of the acclimatization phase on eda and correctness is not worth considering further. Therefore, we consider all triplets for the subsequent analyses unless otherwise specified.

### Q1: There are dependencies among the three quantifications of uncertainty

We used mixed models with random intercepts to identify correlations among Stated_U, duration and EDA for the *n* = 29 annotators. As already described, duration was affected by the acclimatization phase. Hence, we excluded the first three triplets from the statistical analysis in Table 3.

**Table 3:** Statistical analysis on the association between Stated_U and duration for each triplet annotation task (first row), between EDA and duration (second row) and between EDA and Stated_U (last row). The association between Stated_U and duration is significant.

exposure	outcome	ß (95%-Confidence Interval)	p
Stated_U	duration	6.46 (3.34; 9.58)	<0.001
EDA	duration	9.46 (-7.46; 26.40)	0.273
Stated_U	EDA	-0.00 (-0.01; 0.00)	0.294

Table 3 shows that there is a significant association between duration and Stated_U, meaning that with increased Stated_U annotators needed more time to decide. However, we observed no significant association of eda to Stated_U, nor to duration. In contrast, the stress levels recorded with electrodermal activity do not agree with how certain the annotators felt about their decisions, nor with the amount of time they used to meet these decisions.

### Q2: Non-experts and experts have comparable correctness

**Fig 4:**
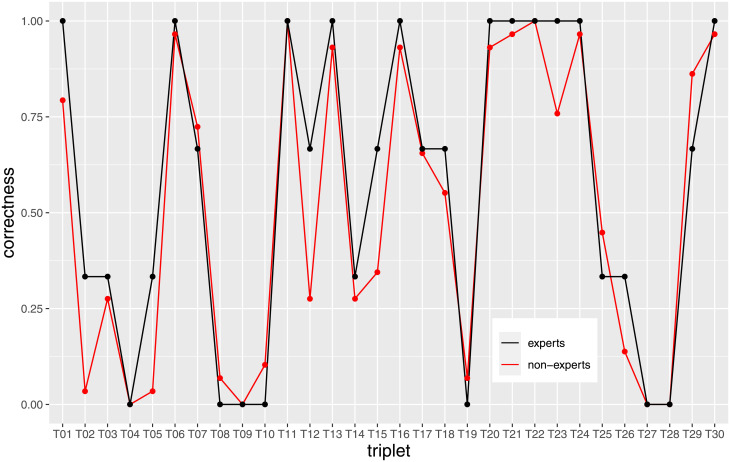
*T_CorrectnessRatio* for the experts (black line) and for the annotators (red line). The two groups performed similarly, and misclassified some triplets in agreement; slight differences can be explained by the large difference between the number of non-experts (29) and the number of experts (3).

We juxtaposed the correctness of the non-expert annotators to that of the three experts. Fig 4, we depicted the T_CorrectnessRatio for each triplet, once for the experts (black line) and once for the non-expert annotators (red line). The plots indicate that the correctness is almost the same. The fluctuations can be explained by the small number of experts in the experiment (3 experts vs 29 non-experts).

### Q2: Associations between uncertainty and correctness

In our investigation, we placed much emphasis to the relationship between the uncertainty indicator Stated_U and the annotator correctness, since Stated_U is a direct feedback delivered by the annotators themselves: when an annotator feels uncertain, then the triplet annotation might be ignored; when an annotator feels certain, then the triplet annotation may be considered further, especially if there is agreement among the annotators. For this purpose, we used visualizations and juxtaposed annotator Stated_U and correctness. Our analysis on how the objective quantities of duration and eda (stress levels) associate to correctness is simpler, using only mixed models with random intercepts.

#### No visible association between uncertainty and annotator correctness

The 29 annotators delivered 870 annotations (for the 30 triplets). For 219 (25.17%) of these annotations, they stated to be very certain, for 317 (36.43%) rather certain, for 194 (22.29%) rather uncertain and for 140 (16.09%) very uncertain. We depicted the relationship between correctness and *binary uncertainty* in Table 4.

**Table 4:** Correctness vs Stated_U, here aggregated into *binary uncertainty*.

	correctness		
	correct	incorrect	Total
*binary uncertainty*= 0 → certain	281	255	536
*binary uncertainty*= 1 → uncertain	156	178	334
Total	437	433	870

We see in Table 4 that the likelihood of being uncertain is higher among the incorrect choices than among the correct ones, and that the likelihood of being correct is higher when one is certain than when one is uncertain. However, this can be explained by the fact that the likelihood of being certain is higher than the likelihood of being uncertain. We see that the likelihood of being correct when one feels uncertain is 46.70% (156/334) and thus lower than the likelihood of being correct when one feels certain (281/530 = 53.01%). However, the likelihood of being incorrect when one feels certain is also rather high (255/536 = 47.57%), since the annotators stated being certain of their choices with a high likelihood of 61.60% (536/870). Indeed, the statistical test (Pearson *χ*^2^) returned a p-value of 0.101, indicating no statistical significance.

**Fig 5:**
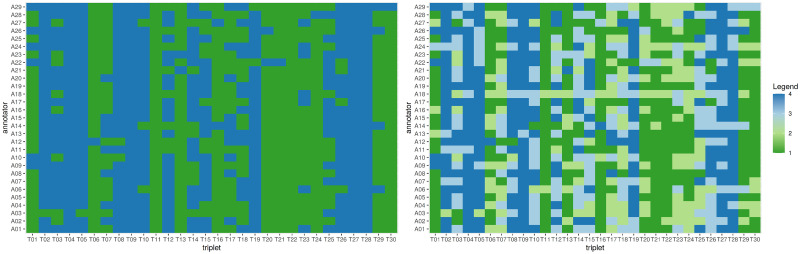
Heatmaps of correctness and Stated_U and correctness. Left subfigure—Heatmap of correctness: green indicates correct annotation of a triplet (x-axis) by an annotator (y-axis), while blue indicates incorrect choice (left subfigure). Right subfigure—refined Heatmap of Stated_U and correctness: correct and certain (1: green intensive), correct and uncertain (2: lime green), incorrect and uncertain (3: light blue), incorrect and certain (4: blue intensive). Some triplets are consistently annotated incorrectly by (almost) all annotators.

In the heatmap left subfigure of Fig 5 we captured the influence of each triplet on annotator correctness: green indicates that the annotator annotated the triplet correctly, blue that the annotation was incorrect. The blue columns mean that some triplets were annotated incorrectly by (almost) all annotators.

The heatmap at the right side of Fig 5 captures the relationship between correctness (correct: green, incorrect: blue) and Stated_U (low: intensive color, high: light color). This subfigure still contains several columns in intensive blue, i.e. triplets for which most of the annotators stated low Stated_U but annotated them incorrectly. This means that certainty, as stated by the annotators themselves, does not imply correct annotation.

### Q3: Annotator agreement is not associated with correctness

To assess agreement among annotators, we first studied the overall likelihood of considering entity B as more similar to A (ill) vs more similar to C (healthy). Our results on Table 5 show that C was preferred over A.

**Table 5:** Number of times an annotator has opted for annotation value A vs C, distinguishing between correct and incorrect choice. C is preferred over A.

	correct choice	incorrect choice
A	208	206
C	229	227

The value of Krippendorff’s *α* was 0.593 when considering all triplets, and 0.608 when skipping the first three triplets in response to the findings on acclimatization (cf. Results on Q0). This is lower than .667, which is considered in [29] as “the lowest conceivable limit” (page 429). The *α*-values we computed seem thus to indicate that the annotations were unreliable and, hence, that there was some inherent difficulty in the annotation task per se.

To refine this finding, we next aggregated the annotation values over each triplet and inspected the distribution, using the quantification of agreement in Eq 2 and two threshold values. The results are depicted on Table 6, where the last column shows agreement in 28 out of 30 triplets. This column indicates that the likelihood of agreeing on A is the same as agreeing on C. However, under the more restrictive threshold $\tau =\frac{3}{4}$, the non-expert annotators agree on C in 13 cases and agree on A only in 9 cases.

**Table 6:** Agreement of the annotators under each threshold *τ* on the annotation values A and C, correctly or incorrectly.

	agreement under $\tau =\frac{3}{4}$		agreement under $\tau =\frac{2}{3}$ ONLY		
	correct	incorrect	correct	incorrect	Total
A	5	4	2	3	14
C	7	6	0	1	14

Finally, we inspected the agreement of annotators towards each triplet and juxtaposed it to the correctness towards triplet. The results are depicted on Table 7.

**Table 7:** Juxtaposition of annotator performance (last column) and agreement on triplet for $\tau =\frac{2}{3}$ and $\tau =\frac{3}{4}$. We mark in **bold** the triplets for which experiment participants agreed on the wrong value for both thresholds, and in *italic* the triplets for which experiment participants agreed on the wrong value only for $\tau =\frac{2}{3}$.

	correct	*argm*(*t*)		*agreement*_*τ*_(*t*)		*T_CorrectnessRatio*(*t*)
*t*	A or C	*voteFor*(*t*, *A*)	*voteFor*(*t*, *C*)	$\tau =\frac{2}{3}$	$\tau =\frac{3}{4}$	
01	C	0.21	0.79	1	1	0.79
**02**	C	0.97	0.03	1	1	0.03
*03*	C	0.72	0.28	1	0	0.28
**04**	A	0.00	1.00	1	1	0.00
**05**	A	0.03	0.97	1	1	0.03
06	A	0.97	0.03	1	1	0.97
07	A	0.72	0.28	1	0	0.72
**08**	C	0.93	0.07	1	1	0.07
**09**	C	1.00	0.00	1	1	0.00
**10**	C	0.90	0.10	1	1	0.10
11	A	1.00	0.00	1	1	1.00
*12*	C	0.72	0.28	1	0	0.28
13	C	0.07	0.93	1	1	0.93
*14*	C	0.72	0.28	1	0	0.28
*15*	A	0.34	0.66	1	0	0.34
16	C	0.07	0.93	1	1	0.93
17	A	0.66	0.34	1	0	0.66
18	A	0.55	0.45	0	0	0.55
**19**	A	0.07	0.93	1	1	0.07
20	C	0.07	0.93	1	1	0.93
21	C	0.03	0.97	1	1	0.97
22	C	0.00	1.00	1	1	1.00
23	A	0.76	0.24	1	1	0.76
24	A	0.97	0.03	1	1	0.97
25	C	0.55	0.45	0	0	0.45
**26**	A	0.14	0.86	1	1	0.14
**27**	A	0.00	1.00	1	1	0.00
**28**	A	0.00	1.00	1	1	0.00
29	C	0.14	0.86	1	1	0.86
30	A	0.97	0.03	1	1	0.97

The last column of Table 7 shows the aggregated correctness of the 29 annotators *T*_*CorrectnessRatio* for each triplet *t*, while the previous two columns depict the level of agreement for *t* among the annotators *agreement*_*τ*_(*t*), for two threshold values. To juxtapose correctness with agreement, we show in the second column the correct value for the triplet, i.e. A or C, while the next two columns show the ratio of votes of the annotators for each of A and C. Obviously, if the correct value for triplet *t* is V, then it holds that *T*_*CorrectnessRatio*(*t*) = *voteFor(t, V)*.

On that table, we marked in red the triplets where the annotators agreed for the most restrictive threshold $\tau =\frac{3}{4}$ on the incorrect annotation value (A instead of C, or vice versa), leading in very low aggregate correctness, see e.g. triplet 04. One third of all triplets (10 in total) are so marked. Further, we marked in orange the triplets where the annotators agreed on the incorrect value only under the less restrictive threshold $\tau =\frac{2}{3}$. Hence, almost half of the triplets 14 out of 30) were annotated erroneously by $\tau =\frac{2}{3}$ of the annotators. This implies that high values of agreement did *not* lead to higher correctness.

### Q4: Explaining the relationship between correctness and uncertainty

#### ASBA outperforms the annotators

Since the correctness of the annotators was not very high, we first investigated the limits of correctness by comparing to ASBA: being a piece of software, ASBA could capitalize on numerical computations of similarity much better than the human eye.

For $\text{ASBA}\_U\_{\text{Binary}}_{\tau_{ASBA}}$, we set *τ*_*ASBA*_ = 0.5 (cf. Eq 7), i.e. the prior likelihood of each of the two classes: the classification problem to be solved is binary and balanced; in 50% of the triplets, the middle record is similar to A, in the other 50% it is similar to C.

Table 8 depicts the Stated_U and correctness values of the annotators for each triplet and juxtaposes them to the derived values of ASBA. The correctness of ASBA for one triplet can only be 1.00 (correct annotation) or 0.00 (incorrect annotation). Hence, for the juxtaposition, we use the agreement threshold *τ* as lower boundary to *T*_*CorrectnessRatio*, cf. last three columns of Table 7. The rationale is that if the annotators agree on the correct annotation *V* (one of A, C), then the *T*_*CorrectnessRatio* is equal to *voteFor*(*V*). Accorgingly, in the 4th column of Table 8 we mark with bold and italic all *T*_*CorrectnessRatio* values that are above $\tau =\frac{2}{3}$ and below $\frac{3}{4}$ and with bold all *T*_*CorrectnessRatio* values that are above $\tau =\frac{3}{4}$. In the 5th column, we mark with bold all ASBA values that are equal to 1.0, i.e. correct annotations.

**Table 8:** Juxtaposition of stated uncertainty and correctness of the annotators and the Artificial Similarity-Based Annotator (ASBA) for each triplet.

Triplet ID	annotator Stated_U	uncertainty of ASBA	correctness	
			annotator	ASBA
01	0.30	*0.91*	**0.79**	0.00
02	0.26	*0.93*	0.03	0.00
03	0.63	*0.93*	0.28	0.00
04	0.21	0.44	0.00	**1.00**
05	0.38	*0.60*	0.03	**1.00**
06	0.41	*0.79*	**0.97**	**1.00**
07	0.64	*0.94*	***0.72***	**1.00**
08	0.26	*0.70*	0.07	0.00
09	0.15	0.35	0.00	**1.00**
10	0.55	*0.69*	0.10	0.00
11	0.09	0.19	**1.00**	**1.00**
12	0.57	*0.89*	0.28	0.00
13	0.34	0.43	**0.93**	**1.00**
14	0.67	*0.63*	0.28	0.00
15	0.60	*0.87*	0.34	**1.00**
16	0.23	*0.59*	**0.93**	**1.00**
17	0.58	*0.97*	***0.66***	**1.00**
18	0.76	*0.90*	0.55	**1.00**
19	0.38	0.33	0.07	**1.00**
20	0.48	*0.58*	**0.93**	**1.00**
21	0.22	0.43	**0.97**	**1.00**
22	0.36	0.45	**1.00**	**1.00**
23	0.71	*0.92*	**0.76**	0.00
24	0.46	0.47	**0.97**	**1.00**
25	0.62	*0.82*	0.45	**1.00**
26	0.57	*0.96*	0.14	0.00
27	0.39	*0.64*	0.00	0.00
28	0.40	*0.74*	0.00	0.00
29	0.39	*0.58*	**0.86**	0.00
30	0.29	*0.77*	**0.97**	**1.00**

When comparing the marked positions in the 4th and 5th column of Table 8, we see that ASBA performed a correct annotation for 18/30 triplets, while the group of annotators agreed on the correct annotation for 12/30 triplets under $\tau =\frac{3}{4}$ and for 3 additional triplets under $\tau =\frac{2}{3}$, resulting in a ratio of 15/30 total. Hence, ASBA is slightly superior to the annotators’ group.

The juxtaposition of Stated_U of the annotators to $\text{ASBA}\_U\_{\text{Binary}}_{\tau_{ASBA}}$ is on the 2nd and 3rd column of Table 8. Italic values indicates annotations performed by ASBA with higher uncertainty than *τ*_*ASBA*_. All 8 triplets that were annotated with low uncertainty by ASBA were correctly annotated. Notably, the three triplets #04, #09 and #19 which were incorrectly annotated by the annotators despite low uncertainty were all correctly annotated by ASBA, again with low uncertainty. Hence, for ASBA low uncertainty implies a correct annotation and can be relied upon.

**Fig 6:**
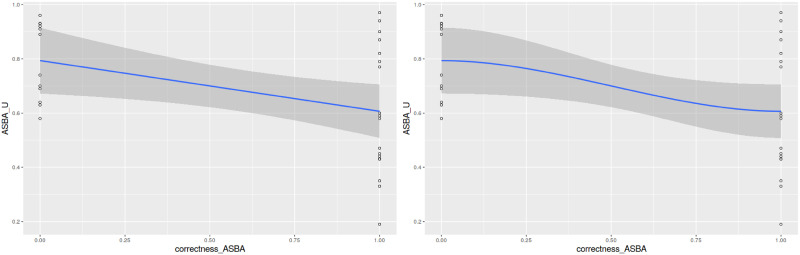
Relationship between ASBA correctness and uncertainy of ASBA using linear regression (left subfigure) and LOESS regression (right subfigure). The correctness increases as uncertainty decreases.

We used regression to capture the relationship between ASBA correctness and the values of ASBA_U (Fig 6). Both the linear regression (left subfigure) and the LOESS regression (right subfigure) show a clear downward trend, indicating that correctness improves as uncertainty decreases.

#### The order of the triplets affects annotation duration but not EDA

The results of Table 7 on agreement among annotators and of Table 8 on uncertainty of annotators and of ASBA indicate that there are triplets for which the annotators are certain and agree on an erroneous annotation, while ASBA is uncertain.

On Table 9, we see the relationship between Triplet ID and the observed forms of uncertainty, namely duration (upper part of the Table) and eda (lower part), using a mixed model with random intercept. The effect of Triplet ID on duration is highly significant at the confidence level we used, namely 95%, but also at 99%. The coefficient -0.71 (seconds) indicates that the annotators’ annotation speed increased as they progressed from the first to the last triplet (triplets were seen in order of ID). In contrast, EDA values are not associated to Triplet ID: since EDA captures stress levels, this means that the stress level during the annotation of a triplet is not modified by the ordinal position of the triplet.

**Table 9:** Associations of Triplet ID with duration and EDA. Associations were analyzed by mixed models with random intercept.

exposure	outcome	ß (95%—Confidence Interval)	p
Triplet ID	duration	-0.71 (-0.89; -0.53)	<0.001
Triplet ID	EDA	-0.00 (-0.00; 0.00)	0.221

**Fig 7:**
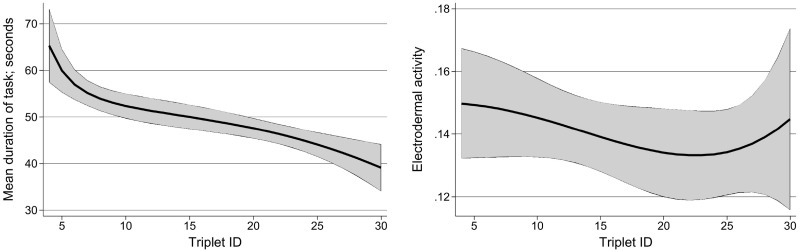
Relationship between Triplet ID and duration and EDA. Modelling using fractional polynomials with non-linear progressions for duration (left subfigure) and for EDA (right subfigure).

The results on the two Tables are further refined on Fig 7. The left subfigure captures the correlation between duration and Triplet ID visually. The right subfigure shows that EDA does *not* drop with Triplet ID and actually increases for the last few triplets. When juxtaposing this increase to the agreement and correctness of the annotators for the last triplets (cf. Table 7), we see that three out of the five last triplets were annotated incorrectly and with large agreement. Hence, the increase in stress levels seems to reflect the erroneous annotations, while the Stated_U does not (cf. 2nd column of Table 8, last triplets).

These results indicate that duration is not an appropriate indicator of uncertainty, while the stress levels (in the form of electrodermal activity) may capture an uncertainty that is not explicitly stated.

#### The interplay of correctness and uncertainty

In Table 10 we investigated associations of correctness with duration, EDA and Stated_U. For these analyses we again skipped the first three triplets, because of the effect of the acclimatization phase on duration (cf. Results on Q0). All these analyses were adjusted for Triplet ID, because the Triplet ID was considered as a confounder for the associations of correctness with duration, EDA and Stated_U. We observed no significant associations of correctness with duration or EDA. On the other hand Stated_U was positively associated with correctness. Likewise, the Triplet ID showed a positive association with correctness.

**Table 10:** Associations of correctness with duration, EDA and Stated_U. Associations were analyzed by mixed models with random intercept.

exposure	outcome	ß (95%—Confidence Interval)	p
correctness	duration	-0.31 (-3.14; 2.51)	0.827
Triplet ID		-0.70 (-0.89; -0.52)	<0.001
correctness	EDA	0.00 (-0.00; 0.01)	0.536
Triplet ID		-0.00 (-0.00; 0.00)	0.189
Stated_U	correctness	0.74 (0.55; 0.99)	0.046
Triplet ID		1.04 (1.02; 1.06)	<0.001

In Table 11, we show the association between uncertainty as stated (Stated_U) and uncertainty as observed (duration, eda) when controlling for Triplet ID and after skipping the first three triplets of the acclimatization phase. These results extend those on Table 3 by highlighting the direction of the correlation to the ordinal number of the triplet.

**Table 11:** Associations of Stated_U with duration and EDA. Associations were analyzed by mixed models with random intercept.

exposure	outcome	ß (95%—Confidence Interval)	p
Stated_U	duration	7.10 (4.11; 10.09)	<0.001
Triplet ID		-0.73 (-0.91; -0.55)	<0.001
Stated_U	EDA	-0.00 (-0.01; 0.00)	0.322
Triplet ID		-0.00 (-0.00; 0.00)	0.242

We observed a significant positive association of Statated_U with duration indicating that with increasing uncertainty the annotators worked longer on a task. With increasing Triplet ID the duration for a task decreased significantly. This is in line with the results we showed in the heatmap shown in the right subfigure of Fig 5. This where light blue and light green colors indicate uncertainty and are more rare in the left part of the map (lower ordinal numbers). The second column of Table 8 confirms that indeed the Stated_U values for the first 10 triplets are mostly low, the increase starts after triplet #11. The third column of Table 8 depicts the ASBA_U, an objective measure that shows how close all three instances of a triplet are to each other and thus how difficult it is to decide which two are most similar: the first 10 triplets were not easier to annotate than the subsequent ones. Hence, the correlation between duration and Stated_U when controlled by Triplet ID and in juxtaposition to the uncertainty and correctness of ASBA indicate a spurious relationship that manifests itself in the first third of the experiment and wanes only partially afterwards, as Stated_U increases but duration keeps decreasing.

The first row of the lower part of Table 11 shows that there is no significant relationship between eda and Stated_U. This can be explained by the large variance of eda itself, as can be seen in the wide gray ribbon of standard deviation around the fractional polynomial in the right subfigure of Fig 7. This polynomial is close to a straight line (cf. vertical axis), but the magnification indicates the role of the triplet ordinal number: the eda decreases slightly as the experiment proceeds but increases again at the end. The Triplet ID as ordinal number does not *and should not* have an effect on eda, since the triplets with small ordinal numbers are not easier or more difficult than those with large ordinal numbers.

## Discussion

### Discussion of the findings

We investigated the interplay of annotator correctness and annotator uncertainty and agreement, and we searched for factors that can explain this interplay. We distinguished between uncertainty as stated by each annotator and uncertainty as observed, in the form of annotation duration and in the form of stress levels during annotation. We found that neither annotator agreement nor uncertainty could explain correctness. In contrast, there were incorrect annotations met with almost unanimous agreement and very low stated uncertainty. Annotation duration was found to be correlated to stated uncertainty, and this correlation became significant when taking the ordinal number of the annotation tasks into account. Our findings agree only partially with earlier findings in the literature.

In [31], Cocos et al. found that “inter-annotator agreement among the crowdsourced workers was lower than agreement between [the] expert annotators.” We had only three experts in our experiment, so agreement among them could not be quantified in a robust way. However, we compared their correctness to that of the non-expert annotators and found them comparable.

In [32], Hata et al. found that “workers are extremely stable in their quality over the entire period”. We can only confirm this to a limited extent, because the correctness of the annotators was low in general and lower than that of the mechanistic annotator ASBA.

Ahonen et al. [33] have measured EDA in an experiment on identifying similar pairs within triplets but did not come to conclusive results. We also found that EDA is not predictive by itself, but we still found associations between correctness and uncertainty indicators. The task of identifying the most similar pair inside a triplet of images has been investigated in [14] and in [33], whereby Ahonen et al. [33] have also measured EDA in their experiment. Ahonen et al. [33] did not come to conclusive results on the predictiveness of EDA.

High agreement is widely used as indicator of high correctness [34]. When studying agreement in combination with uncertainty for individual triplets, we found that some triplets were erroneously annotated with high agreement and low uncertainty, and this was done by both experts and non-experts. This indicates that there is an *inherent* property of the tasks, a kind of *difficulty* or *hardness-to-solve*, which may mislead the annotators. The indication is also supported by the correctness of ASBA, whose mechanistic nature allowed it to exploit similarity in a high-dimensional space better than a human could. Since inherent properties cannot be known in advance, the reliability of high agreement should be questioned.

Literature has proposed lower boundaries to the agreement measurement function ‘Krippendorff’s *α*’, i.e. to the level of agreement demanded in order to reach at least ‘tentative conclusions’ [29]. The level of agreement among the annotators in our experiment was lower than the lowest of these boundaries, implying that the annotations should not be relied upon. This might be interpreted as an indication that Krippendorff’s *α* can predict the aforementioned inherent difficulty or hardness-to-solve. However, this interpretation is not well secured, for following reason. Annotation reliability may be associated to annotator skills as well as task difficulty. In our controlled experiment, all annotators had the same skills with respect to the annotation task. When neither the annotator skills nor the difficulty of the task design per se (as opposed to the difficulty of one of the individual tasks) are known, more research is needed to assess whether Krippendorff’s *α* can discern the objective difficulty of a fully unknown task that has no ground truth.

Our experiment confirmed a correlation between annotation duration and stated uncertainty. However, we also found a significant negative correlation between duration and task ordinal position, showing that the annotators became faster with time. This might indicate that they were forming a mental model of the tasks, which allowed them to be faster, but did not make them perform better. Also, stated uncertainty increased with time. Hence, the relationship between annotation duration and stated uncertainty across the sequence of annotation tasks needs further investigation.

Gadiraju et al. [34] introduced a concept of objective task difficulty. For example, in an experiment on the transcription of captcha images, they considered captchas of increasing difficulty. However, in that experiment, the difficulty was known, while in our experiments it was not. In a real crowdsourcing assignment, the level of difficulty for each individual task cannot be known in advance.

Task duration has often been suggested as indicator of difficulty [34–36]. We could not capture difficulty but we measured uncertainty and we found correlations between duration and stated uncertainty. However, we also found an association between duration of each task and ordinal number of task. Hence, it turned that duration is affected by further factors and therefore should not be assumed to predict difficulty.

Inherent task difficulty in crowdworking was investigated by Raebiger et al. [37], albeit they used crowdworker disagreement as indication of inherently hard tasks. We found that disagreement is not a good indicator to this purpose. However, our experiment does confirm that *there are* inherently hard tasks. Our statistical results indicate that this inherent property might be a confounder that explains the unexpected relationship among duration, uncertainty and task ordinal number—in the sense that the annotators might have realized with time that some of the tasks were more hard than they thought. The experiment participants were representative in terms of medical background knowledge. They were indeed not representative in terms of age, and they were likely to be better in pattern recognition than crowdworkers in general. Also, their engagement was very high: this was mission-critical, since we wanted to assess task difficulty.

Jambigi et al. [38] created a workflow where the association of electrodermal activity with task difficulty was established inside a pre-experiment; the time series classifier thus designed was able to separate between easy and difficult tasks of different nature than in the pre-experiment. In our experiment, task difficulty was not known in advance. However, their approach could be used as a preparatory step to an experiment like ours, in order to identify the stress levels that indicate difficult vs easy tasks.

Our Artificial Similarity-based Annotator ASBA played a key role in interpreting annotator behaviour. ASBA outperformed the human annotators and disagreed with them with respect to uncertainty. Since the mechanistic notion of ASBA uncertainty is based on the similarity among all three instances per triplet in the complete feature space, while the quantifications of annotator uncertainty measure perception, we have an indication that the annotators did not consider all dimensions of the feature space during annotation and might have priorized similarity across some dimensions over others. This issue requires further investigation. In particular, we plan to investigate whether annotators that see less variables than ASBA perform better than ASBA, in the sense that showing too many variables may be distracting.

From the machine learning perspective, the process of content annotation is mission-critical for the proper exploitation of supervised machine learning methods in problems like classification of instances, text characterization and categorization. This process is human-resource intensive and requires expertise in the subject area, in which the annotation takes place. In recent years, crowdsourcing has been used to alleviate the demand of elaborate human expertise [39].

From the medical perspective it is very difficult to determine hepatic steatosis based on ten indicators, since hepatic steatosis is a multifactorial disease and its development is a complex interplay between behavioural, metabolic, inflammatory and genetical factors [40]. Even though we selected well-known predictors for hepatic steatosis as input variables [22], the annotation task presented in this manuscript is challenging even for a medical expert. For mild hepatic steatosis, we previously showed a much lower sensitivity of ultrasound-derived hepatic steatosis than for moderate or severe hepatic steatosis [41]. If two measurement techniques for hepatic steatosis did not agree well, it would be even impossible for annotators. For this reason we excluded all individuals with mild hepatic steatosis before setting up the triplets.

Our findings on the behaviour of the annotators versus ASBA stress the importance of mechanistic simulation in pre-experiment settings. This agrees with the findings of Jager et al. in [42], who proposed an agent-based computer simulation to “identify the problems associated with crowdworking and their causes in order to find ways to steer these self-organizing systems on a course towards a solution that provides access to paid work for many diverse people, while limiting the possibilities for exploitation.” With help of ASBA, we found that the annotators felt more certain about their annotations than the data content permitted. We have shown that a mechanism who perceives similarity in the complete feature space delivers more trustworthy decisions: when ASBA decided with high certainty, the annotation could be trusted; when the annotators decided with high uncertainty, the annotation could still not be trusted. Therefore, we intend to investigate how to involve a mechanistic annotator like ASBA in the work of the human annotators, e.g. as an instrument that varies the set of variables (the subspace) shown to annotators and checks whether annotator groups working on different subspaces are in agreement with each other and whether this kind of agreement predicts correctness. This seems particularly important for medical annotation, where there are many variables and the importance of each one for each data record is not known.

### Limitations to validity

Limitations to our findings come mainly from the visual representation of triplets and from the triplet-comparison task as such.

The visual representation of triplets depends on the nature of the triplets’ contents. In the study of [13] and in the juxtaposition of the two pairs within each triplet in [33], the instances to be compared are images. In the experiment of Jambigi et al. [38], the triplets were either texts or images. In our experiment, the instances were rather abstract representations, namely the answers of the participants of an epidemiological study to questionnaires: some of whose features were of numerical nature while others were character strings. This made the comparison challenging.

Triplet-based learning is increasingly popular under machine learning, see e.g. [43–46]. Such studies focus on assessing similarity without human intervention. Evidently, and as supported by the performance of ASBA, humans have more difficulties in perceiving a large feature space than a machine has. Thus, the triplet annotation problem was difficult. It is possible that for easier tasks, agreement and uncertainty predict performance quality better. However, the inherent difficult of a specific triplet cannot be assessed in advance, except for extreme cases, e.g. when the three instances are identical.

We selected triplet annotation as paradigm for the characterization of medical records. In [47], Nissim et al. used medical experts to create a ground truth for their active learning system CAESAR, and they showed that active learning can be used to build classifiers with fewer data and less costs. However, medical experts for a specific disease are an expensive human resource. On the other hand, crowdworkers lack the knowledge needed to perform diagnostic procedures. Hence, the experiment participants of our annotation experiment might have performed better if they were familiar with the diagnostics of this very specific disease. However, crowdworking is intended for applications that do not demand high expertise and specialization.

The experimental setting involved the use of a sensor placed in the palm of each experiment participant’s not dominant hand. This may have lead to a stress level increase by itself and thus explain why electrodermal activity did not predict correctness. However, correctness was low anyway and may have been affected by the inherent but unknown difficulty of the individual triplets. The experiments of Jambigi et al. [38] indicate that the sensors are sensitive enough to capture the difference between easy and difficult tasks, when task difficulty was known in advance.

Some of the above limitations can be taken over as lessons learned when designing crowdworking tasks. (a) A preparatory phase seems essential, to verify whether the crowdworking task is indeed appropriate for crowdworkers or should rather be confined to software that can exploit large feature spaces and numerical differences better than humans. Machine learning can bring major advantage in that context. (b) The visual representation of abstract objects must be as intuitive and informative to the crowdworkers as possible. Research is needed to identify representation forms that help crowdworkers without suggesting that some dimensions are more important than others. (c) It is useful to conduct pre-experiments that assess the difficulty of individual tasks, in order to avoid sending to the crowdworkers tasks for which agreement and uncertainty are not predictive of performance. Ultimately, inherent task difficulty cannot be assessed in all cases, but it is worth looking for confounders in the relationship between uncertainty and performance and between agreement and performance. In some cases, the confounder may be in the representation, which can be controlled and improved. Our proposed workflow and experiment design can be used to expose groups of annotators to different representations and to check whether agreement *among representations* is predictive of correctness.

## Supporting information

S1 File Overview of notation. (PDF)

S2 File Details on the impact of the first three triplets (Q0). (PDF)

S3 File Additional measurements concerning stated uncertainty and stress levels of the annotators (Q1). (PDF)

S4 File Uncertainty quantifications vs correctness, without controlling on Triplet ID (Q2). (PDF)

S5 File Juxtaposition of experts and annotators on correctness (Q2). (PDF)

S6 File Juxtaposition of annotators and ASBA on correctness and uncertainty (Q4). (PDF)

S7 File Impact of configuration (Q4). (PDF)

S8 File End-of-study questionnaire. (PDF)

S1 Dataset Participant answers. (CSV)

S2 Dataset EDA. (CSV)

S3 Dataset End-of-study questionnaire answer. (CSV)
