# Supplementary material for: Assessing the difficulty of annotating medical data in crowdworking with help of experiments
Source: PLoS One. 2021 Jul 29;16(7):e0254764. doi: 10.1371/journal.pone.0254764 (PMC8321104; doi:10.1371/journal.pone.0254764)
Supplement: S1 File — (PDF) [file pone.0254764.s001.pdf]

S1 File: Overview of notation

On S1 Table, we show the core symbols and functions we used in this study.

Symbol	Denotes
x	Annotator
t	Triplet
n	number of annotators
τ	threshold used on annotator agreement and for the binarization of correctness achieved by annotators
**Function**	**Description**
$agreement_{\tau}(t)$	Defined on the number of votes casted for triplet t by the annotators, cf. Eq. 1 and Eq. 2
$A\_correctness(x)$	Number of correct annotations achieved by x and accordingly $A\_correctnessRatio(x)$ over all triplets
$T\_correctness(t)$	Number of correct annotations for t and accordingly $T\_correctnessRatio(t)$ over all annotators; $T\_correctness\_Binary\_\tau(t)$: marks whether t was mostly annotated as correct or not; cf. Eq. 3
$duration(x, t)$	Elapsed time needed by x to annotate t
$eda(x, t)$	Average electrodermal activity for x during the annotation of t; signal is sampled every 10 seconds, hence the number of samples depends on $duration(x, t)$
$Stated\_U(x, t)$	uncertainty of x when annotating t, with four values; $A\_Stated\_U(x)$: sum of Stated_U values for all triplets annotated by x, and $A\_Stated\_URatio(x)$ accordingly; $T\_Stated\_U(t)$: sum of Stated_U values for all annotators who annotated t, and $T\_Stated\_URatio(t)$ accordingly<br><br>$Stated\_U\_Binary(x, t)$: binarization into "low Stated_U" (original values 0 and 1) and "high Stated_U" (original values 2 and 3)
$ASBA\_U(t)$	Uncertainty of our Artificial Similarity-Based Annotator (ASBA) for triplet t computed on how close to each other are the three records to be compared, cf. Eq. 5; $ASBA\_U\_Aggregated(t))$: aggregation into four values (cf. Eq.6), so that it can be compared to $T\_Stated\_URatio(t)$;<br><br>$ASBA\_U\_Binary_{\tau_{ASBA}}(t)$: binarization into two values (cf. Eq.7), so that it can be compared to the binarization of $T\_Stated\_U(t)$

S1 Table: Overview of terms and functions in alphabetical order
