# Supplementary material for: Assessing the difficulty of annotating medical data in crowdworking with help of experiments
Source: PLoS One. 2021 Jul 29;16(7):e0254764. doi: 10.1371/journal.pone.0254764 (PMC8321104; doi:10.1371/journal.pone.0254764)
Supplement: S2 File — (PDF) [file pone.0254764.s002.pdf]

S2 File: Details on the impact of the first three triplets (Q0)

On S2 Table and S3 Table we show the impact of the acclimatisation phase for each annotator on duration (median, harmonic mean), uncertainty, accuracy and correctness.

ParticipantID	duration (sec.) median	harmonic mean	uncertainty	accuracy	correctness
01	59.39	60.38	1	0.44	12
02	45.84	46.99	1	0.48	13
03	37.42	34.01	2	0.63	17
04	57.16	57.41	2	0.56	15
05	72.16	72.67	1	0.52	14
06	19.77	20.49	2	0.52	14
07	52.82	49.71	1	0.52	14
08	33.06	33.05	1	0.52	14
09	49.90	50.49	2	0.56	15
10	26.33	25.05	1	0.56	15
11	49.34	41.16	1	0.59	16
12	21.40	20.30	1	0.56	15
13	20.84	19.82	1	0.44	12
14	39.89	41.91	1	0.48	13
15	55.67	56.36	1	0.44	12
16	97.18	94.32	1	0.48	13
17	76.47	74.68	1	0.56	15
18	34.71	31.49	2	0.52	14
19	52.54	50.22	0	0.48	13
20	41.84	42.21	2	0.44	12
21	40.57	38.44	1	0.59	16
22	53.96	55.40	1	0.52	14
23	54.53	52.42	1	0.44	12
24	48.66	52.53	2	0.52	14
25	37.22	34.63	1	0.52	14
26	29.69	26.68	0	0.56	15
27	22.23	21.33	1	0.56	14
28	43.17	43.56	1	0.48	13
29	26.21	24.37	1	0.52	14

S2 Table: Impact of the first three triplets on uncertainty: Overview of the uncertainty values (lower values indicate higher certainty) for each experiment participant, aggregated over 27 triplets, i.e. after removing the first three ones, juxtaposed to accuracy and correctness, i.e. correct guesses.

ParticipantID	duration (sec.) median	harmonic mean	uncertainty	accuracy	correctness
01	60.93	64.31	1	0.43	13
02	49.62	50.00	1	0.50	15
03	38.61	36.33	2	0.67	20
04	61.97	60.91	2	0.53	16
05	76.86	75.84	1	0.50	15
06	21.32	21.34	2	0.50	15
07	54.10	52.86	1	0.50	15
08	34.43	34.59	1	0.50	15
09	51.15	53.06	2	0.50	15
10	26.91	26.50	1	0.53	16
11	50.35	43.81	1	0.57	17
12	22.81	21.21	1	0.53	16
13	22.83	20.92	1	0.43	13
14	41.13	44.26	1	0.47	14
15	56.38	58.56	1	0.43	13
16	99.76	97.29	1	0.50	15
17	80.53	78.52	1	0.53	16
18	35.76	33.92	2	0.53	16
19	54.64	52.85	0	0.47	14
20	45.24	45.04	2	0.43	13
21	40.10	39.26	1	0.57	17
22	56.80	59.38	1	0.50	15
23	56.74	56.04	1	0.47	14
24	54.02	56.35	2	0.47	14
25	39.39	37.17	1	0.50	15
26	29.92	28.47	0	0.50	15
27	24.54	22.29	1	0.57	17
28	43.73	46.28	1	0.47	14
29	26.26	25.77	1	0.47	14

S3 Table: Overview of the uncertainty values (lower values indicate higher certainty) for each experiment participant, aggregated over all 30 triplets, juxtaposed to accuracy and correctness, i.e. correct guesses
