# Supplementary material for: Assessing the difficulty of annotating medical data in crowdworking with help of experiments
Source: PLoS One. 2021 Jul 29;16(7):e0254764. doi: 10.1371/journal.pone.0254764 (PMC8321104; doi:10.1371/journal.pone.0254764)
Supplement: S3 File — (PDF) [file pone.0254764.s003.pdf]

S3 File: Additional measurements concerning stated uncertainty and stress levels of the annotators (Q1)

S3.1. Distribution of stated uncertainty values among the annotators

We depicted the distribution of the four values for stated uncertainty Stated_U on S1 Fig. Each column shows the number of times the corresponding value was chosen, aggregating over all annotators and triplets. Evidently, the likelihood of being certain or rather certain is higher than the likelihood of being rather uncertain or very uncertain.

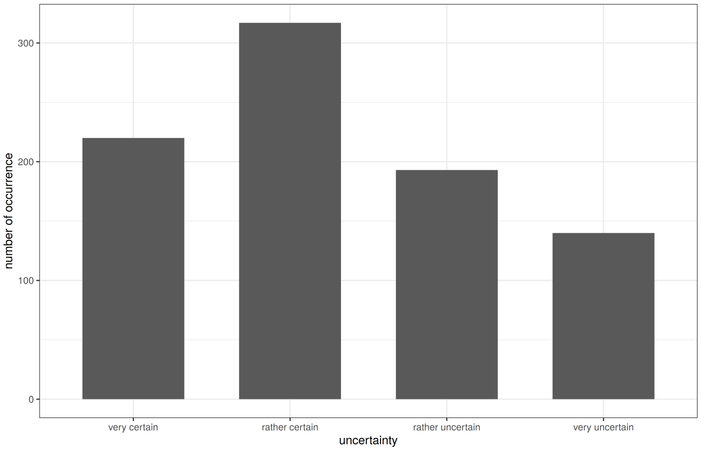

S1 Fig: Sum of the Stated_U for all triplets under specification of each experiment participant. For Stated_U: very certain: 220, rather certain: 317, rather uncertain: 193, very uncertain: 140. For uncertainty_binary: certain: 537, uncertain: 333

On S2 Fig, we depict the stated uncertainty values of the annotators in two heatmaps - on Stated_U with the original four values, and then binarized into 0 (certain: green) and 1 (uncertain: blue).

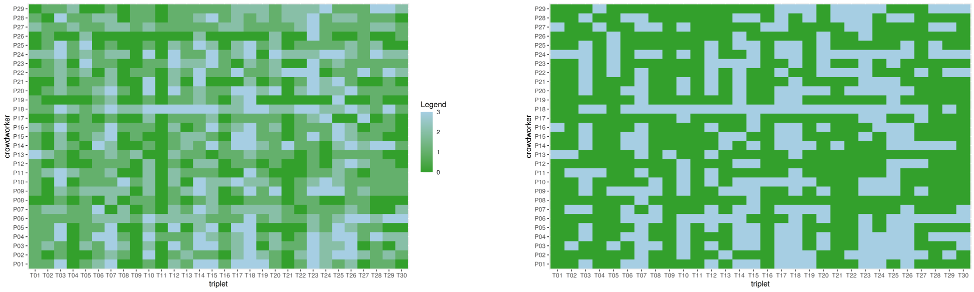

S2 Fig: Stated_U (left subfigure) and uncertainty_binary (right subfigure) - blue is uncertainty of an experiment participant (crowdworker) for a triplet and green is certain of an experiment participant (crowdworker) for a triplet.

S3.2. EDA of each annotators

When giving feedback on the experiment, 3 of the 29 annotators pointed out that the sensor has disturbed them. We have therefore computed a boxplot of EDA, with one

box per annotator, accommodating their EDA values for all 30 triplets. As can be seen in S3 Fig, there were many differences among the values of the annotators, but there were no three annotators with substantially different EDA recordings. Rather, the differences among the annotators can be explained by the electrodermal physiology of the annotators, and by the substantial differences in the time each annotator needed for annotations in general.

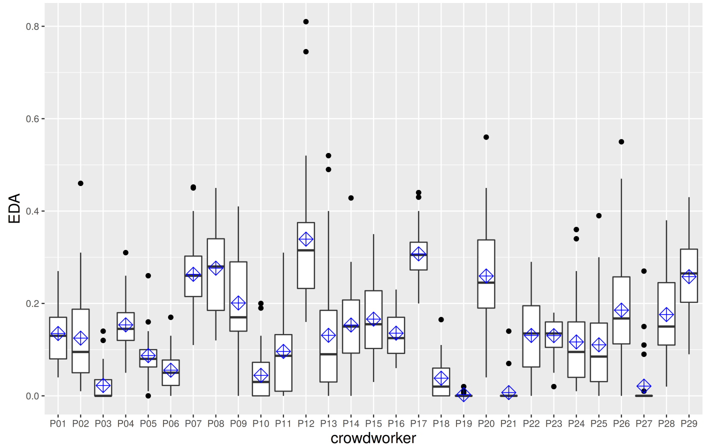

S3 Figure: Boxplot of the EDA values of the experiment participant (crowdworker) over all triplets
