# Supplementary material for: Assessing the difficulty of annotating medical data in crowdworking with help of experiments
Source: PLoS One. 2021 Jul 29;16(7):e0254764. doi: 10.1371/journal.pone.0254764 (PMC8321104; doi:10.1371/journal.pone.0254764)
Supplement: S4 File — (PDF) [file pone.0254764.s004.pdf]

S4 File: Uncertainty quantifications vs correctness, without controlling on Triplet ID (Q2)

We applied mixed models with random intercepts to identify associations between correctness and the three uncertainty quantifications, again skipping the first three triplets because of the effects of the acclimatization phase on duration, one of the three indicators. The results are on S4 Table.

exposure	outcome	β (95% - Confidence Interval)	p
correctness	duration	-2.25 (-5.14; 0.64)	0.128
correctness	EDA	0.00 (-0.00; 0.01)	0.692
Stated_U	correctness	0.77 (0.58; 1.03)	0.084

S4 Table: Statistical analysis on the association between correctness and duration, between correctness and EDA, between Stated_U and correctness: No significant association between: duration and correctness; eda and correctness; correctness and Stated_U.

As we can see on S4 Table, there is a weak associations between Stated_U and correctness (last two lines of the table), but it should be taken with caution, because we did not correct for multiple testing. The other two indicators of uncertainty deliver no associations either. This means that the annotators may err in their annotations without stating that they are uncertain (Stated_U indicator), without taking more time for the annotations (duration indicator) and without increase in their stress levels (eda indicator).
