# Supplementary material for: Assessing the difficulty of annotating medical data in crowdworking with help of experiments
Source: PLoS One. 2021 Jul 29;16(7):e0254764. doi: 10.1371/journal.pone.0254764 (PMC8321104; doi:10.1371/journal.pone.0254764)
Supplement: S5 File — (PDF) [file pone.0254764.s005.pdf]

S5 File: Juxtaposition of experts and non-experts on correctness (Q2)

On S5 Table we show that the correctness of the experts is similar to that of non-experts, ranging between 0.4 and 6, and that it is not much influenced by the acclimatization phase.

expert	performance	remove 1st triplets
01	0.63	0.63
02	0.57	0.56
03	0.50	0.52

S5 Table: Performance of experts for all triplets and without the three first triplets

The map on Fig 4 captures the correct annotations (gray cells) and the incorrect ones (black cells) as given by the experts. When we juxtapose this map to that of Fig 5 for annotators, we see that the tendencies in the annotation of the triplets are largely the same. There are triplets, e.g. 04, 09 and 27, which have been unanimously annotated incorrectly by both experts and annotators.

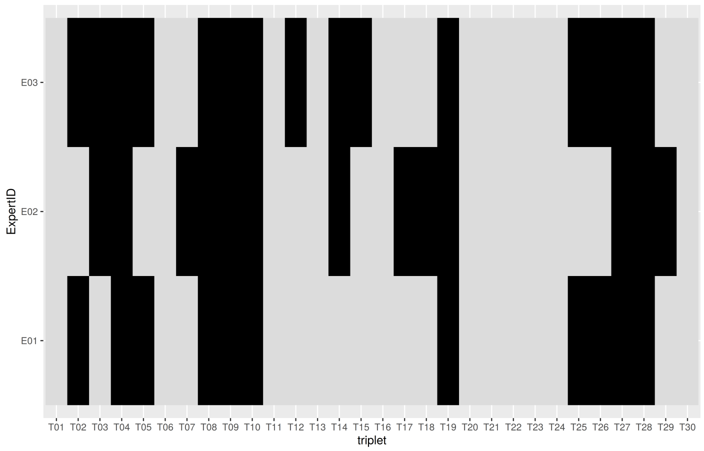

S4 Figure: Heatmap for correctness of the experts: grey is correctness of a expert for a triplet and black is incorrect of a expert for a triplet
