# Supplementary material for: Assessing the difficulty of annotating medical data in crowdworking with help of experiments
Source: PLoS One. 2021 Jul 29;16(7):e0254764. doi: 10.1371/journal.pone.0254764 (PMC8321104; doi:10.1371/journal.pone.0254764)
Supplement: S6 File — (PDF) [file pone.0254764.s006.pdf]

S6 File: Juxtaposition of annotators and ASBA on correctness and uncertainty (Q4)

We juxtaposed the uncertainty and correctness of ASBA per triplet to the stated uncertainty and correctness of the annotators per triplet. The heatmaps are depicted in the S5 Fig , where it must be noted that the combination of incorrect and certain does not occur for ASBA.

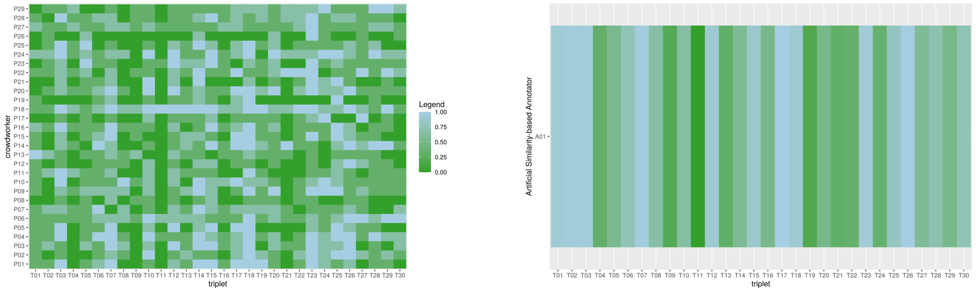

S5 Figure: To compare the uncertainty of a triplet with ASBA, we computed ASBA_U between 0 (very certain) and 1 (very uncertain) based on the four values of Stated_U(left subfigure).
Furthermore we computed the uncertainty for ASBA based on the HEOM distance and divided the smallest distance from the largest distance (right subfigure)

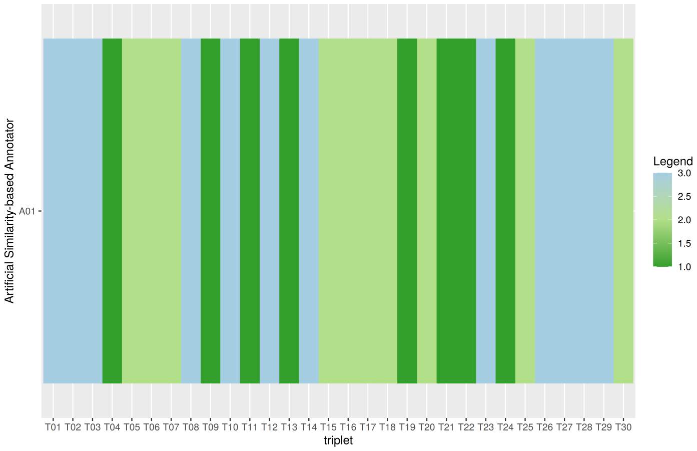

S6 Figure: Heatmap for $ASBA\_U\_Binary_{\tau_{ASBA}}(\cdot)$ and correctness - decision of ASBA for a triplet: correct and certain (1: green intensive), correct and uncertain (2: lime green), incorrect and uncertain (3: light blue), but ASBA is not incorrect and certain
