# Supplementary material for: Assessing the difficulty of annotating medical data in crowdworking with help of experiments
Source: PLoS One. 2021 Jul 29;16(7):e0254764. doi: 10.1371/journal.pone.0254764 (PMC8321104; doi:10.1371/journal.pone.0254764)
Supplement: S7 File — (PDF) [file pone.0254764.s007.pdf]

## S7 File: Impact of configuration (Q4)

Based on the information from the questionnaire, 24 of the 29 annotators (82.76%) preferred a configuration for the decision, which should contribute to the assignment of instance A or instance C. 5 of the 29 annotators (17.24%) did not prefer a configuration. Out of the 24 annotators, 11 annotators (37.93%) preferred the tile based configuration and 13 annotators (44.83%) preferred the, black and white, parallel based configuration.

Did the annotators who preferred one configuration perform differently from those who preferred none? We cannot say. Therefore, we only state that there is no winner configuration.

### S7.1. The annotators preferred answer C (Q4)

We counted annotator correctness for each choice A vs C and investigated whether the one choice was preferred.

As shown on S6 Table, there is a total of 456 answers for C, out of 870, i.e. there is a slight preference of C over A.

| decision - correctness - Stated_U | number of answers |
|---|---|
| C - true - very certain | 64 |
| C - true - rather certain | 98 |
| C - true - rather uncertain | 42 |
| C - true - very uncertain | 25 |
| C - false - very certain | 43 |
| C - false - rather certain | 91 |
| C - false - rather uncertain | 51 |
| C - false - very uncertain | 42 |
| A - true - very certain | 58 |
| A - true - rather certain | 62 |
| A - true - rather uncertain | 47 |
| A - true - very uncertain | 41 |
| A - false - very certain | 54 |
| A - false - rather certain | 67 |
| A - false - rather uncertain | 53 |
| A - false - very uncertain | 32 |

S6 Table: Based on Stated_U and given answers we combine the decisions
