# Supplementary material for: Assessing the difficulty of annotating medical data in crowdworking with help of experiments
Source: PLoS One. 2021 Jul 29;16(7):e0254764. doi: 10.1371/journal.pone.0254764 (PMC8321104; doi:10.1371/journal.pone.0254764)
Supplement: S8 File — (PDF) [file pone.0254764.s008.pdf]

Teil A: Angaben zur Person

Im Folgenden werden zunächst einige Daten zu Ihrer Person erhoben. Diese Daten werden für die statistische Auswertung dieser Studie benötigt. Durch die Beantwortung dieser Fragen kann nicht auf Ihre Person zurückgeschlossen werden.

A1. Bitte geben Sie ihr Alter an.

A2. Bitte geben Sie ihr Geschlecht an.

weiblich ☐

männlich ☐

A3. Bitte geben Sie ihren Studiengang an.

A4. Bitte geben Sie ihre Muttersprache an.

A5. Bitte geben Sie ihr Herkunftsland an.

A6. Sind Sie Links- oder Rechtshänder?

Linkshänder ☐

Rechtshänder ☐

A7. Wie viel Erfahrung haben Sie in folgenden Bereichen?

	keine	wenig	viel	sehr viel
Medizin	☐	☐	☐	☐
Data Mining	☐	☐	☐	☐
Bildverarbeitung	☐	☐	☐	☐

Teil B: Fragen zu der benutzten grafischen Darstellung

Im Folgenden werden Aussagen zu der vorher benutzten grafischen Darstellung aufgelistet.

Bitte kreuzen Sie die Aussagen an, denen Sie zustimmen. Es sind mehrere Ankreuzungen möglich.

B1. Folgende Fragen beziehen sich nur auf die tile-basierte Konfiguration.

	tile-basierte Konfiguration
Die grafische Darstellung war leicht zu verstehen.	☐
Die grafische Darstellung war unnötig komplex.	☐
Die grafische Darstellung war zu überladen.	☐
Die grafische Darstellung war übersichtlich gestaltet.	☐
Die Größe der grafischen Darstellung war angenehm und passend.	☐
Die einzelnen Elemente der grafischen Darstellung waren angenehm groß.	☐
Begriffe und Bezeichnungen der grafischen Darstellung waren leicht verständlich formuliert.	☐
Die grafische Darstellung konnte ich nur mithilfe der Versuchsleiterin verstehen.	☐
Ich halte die grafische Darstellung für nützlich.	☐
Der Umgang mit der grafischen Darstellung fiel mir leicht.	☐
Mithilfe der grafischen Darstellung konnte ich mein Arbeitsziel erreichen.	☐
Mithilfe der grafischen Darstellung konnte ich die Instanzen einfach miteinander vergleichen.	☐
Ich konnte die Instanzen mit Hilfe der grafischen Darstellung schnell miteinander vergleichen.	☐

B2. Folgende Fragen beziehen sich nur auf die parallel-basierte Konfiguration.

	parallel-basierte Konfiguration
Die grafische Darstellung war leicht zu verstehen.	☐
Die grafische Darstellung war unnötig komplex.	☐
Die grafische Darstellung war zu überladen.	☐
Die grafische Darstellung war übersichtlich gestaltet.	☐
Die Größe der grafischen Darstellung war angenehm und passend.	☐
Die einzelnen Elemente der grafischen Darstellung waren angenehm groß.	☐
Begriffe und Bezeichnungen der grafischen Darstellung waren leicht verständlich formuliert.	☐
Die grafische Darstellung konnte ich nur mithilfe der Versuchsleiterin verstehen.	☐
Ich halte die grafische Darstellung für nützlich.	☐

Der Umgang mit der grafischen Darstellung fiel mir leicht. ☐

Mithilfe der grafischen Darstellung konnte ich mein Arbeitsziel erreichen. ☐

Mithilfe der grafischen Darstellung konnte ich die Instanzen einfach miteinander vergleichen. ☐

Ich konnte die Instanzen mit Hilfe der grafischen Darstellung schnell miteinander vergleichen. ☐

B3. **Folgende Frage beziehen sich auf die Kombination der tile- und parallel-basierten Konfiguration.**

Wurde eine Variante bevorzugt für die Entscheidungen

Ja ☐

Nein ☐

B4. **Folgende Frage beziehen sich auf die Kombination der tile- und parallel-basierten Konfiguration.**

Wenn ja, welche?

Tile-basierte Konfiguration ☐

Parallel-basierte Konfiguration ☐

B5. **Sonstige Anmerkungen**

Teil C: Fragen zu der Software

C1. **Im Folgenden werden Aussagen zu der vorher benutzten Software aufgelistet.**

Bitte kreuzen Sie die Aussagen an, denen Sie zustimmen. Es sind mehrere Ankreuzungen möglich.

Die Software war verständlich gestaltet. ☐

Die Software war schwer zu bedienen. ☐

Die Software war gut strukturiert. ☐

Die Software war einfach aufgebaut. ☐

Die einzelnen Elemente der Software waren gut erkennbar. ☐

Die Texte innerhalb der Software waren leicht verständlich formuliert. ☐

Die Größe der Texte war zu klein. ☐

Die verwendeten Begriffe und Bezeichnungen innerhalb der Software waren leicht zu verstehen. ☐

Die Aufgabenstellung war klar formuliert. ☐

C2. Wurde der Aktivitätssensor als Störung während des Experimentes empfunden?

Ja ☐

Nein ☐

Teil D: Feedback

D1. Möglichkeit für Kritik, Lob und Anregungen zu der Studie und zu dieser Befragung zu äußern.

Sie sind am Ende der Befragung angekommen.

Vielen Dank für das Ausfüllen des Fragebogens!

Ich hoffe, Sie hatten Spaß an der Studie und ich bedanke mich für Ihre Zeit und Ihre Mühe.

Anne Rother

Part A: Personal data
In the following, we will first collect some personal data about you. These data are needed for the statistical analysis of this study. Your person cannot be identified by answering these questions.

A1. Please enter your age.

A2. Please enter your sex: female – male

A3. Please enter your course of study.

A4. Please enter your native language.

A5. Please enter your country of origin.

A6. Are you left-handed or right-handed?

A7. How much experience do you have in the following areas?
Medicine- none, little, much or very much
Data Mining- none, little, much or very much
Image processing- none, little, much or very much

Part B: Questions about the used graphical representation
The following is a list of statements about the graphical representation used previously. Please check off the statements you agree with. Multiple check marks are possible.

B1. The following questions refer to the tile-based configuration only.
- The graphical representation was easy to understand.
- The graphical representation was unnecessarily complex.
- The graphical representation was too cluttered.
- The graphical representation was clearly arranged.
- The size of the graphical representation was pleasant and appropriate.
- The individual elements of the graphical representation were pleasantly large.
- Terms and designations of the graphical representation were easily understandable.
- I could understand the graphical representation only with the help of the experimenter.
- I consider the graphical representation to be useful.
- It was easy for me to use the graphical representation.
- With the help of the graphical representation I was able to achieve my work goal.
- I could easily compare the instances with the help of the graphical representation.
- I was able to quickly compare instances using the graphical representation.

B2. The following questions refer to the parallel-based configuration only.
- The graphical representation was easy to understand.
- The graphical representation was unnecessarily complex.
- The graphical representation was too cluttered.
- The graphical representation was clearly arranged.
- The size of the graphical representation was pleasant and appropriate.
- The individual elements of the graphical representation were pleasantly large.
- Terms and designations of the graphical representation were easily understandable.
- I could understand the graphical representation only with the help of the experimenter.
- I consider the graphical representation to be useful.
- It was easy for me to use the graphical representation.
- With the help of the graphical representation I was able to achieve my work goal.

- I could easily compare the instances with the help of the graphical representation.
- I was able to quickly compare instances using the graphical representation.

B3. The following question refers to the combination of tile- and parallel-based configuration. Was one variant preferred for the decisions
yes or no

B4: If yes, which?
tile-based configuration or parallel-based configuration

B5. Other comments

Part C: Questions about the software

C1. The following is a list of statements about the software you used previously.
Please check off the statements you agree with. There are several check marks are possible.

- The software was designed to be understandable.
- The software was difficult to use.
- The software was well structured.
- The software was simply structured.
- The individual elements of the software were easily recognizable.
- The texts within the software were easily understandable.The size of the texts was too small.
- The terms and designations used within the software were easy to understand.
- The task was clearly formulated.

C2. Was the activity sensor perceived as a disturbance during the experiment?
yes or no

Part D: Feedback
D1. Opportunity for criticism, praise and suggestions about the study and this survey.

You have reached the end of the survey.
Thank you for completing the questionnaire!
I hope you enjoyed the study and I thank you for your time and your effort.
